# Enhanced Biocompatibility by Evaluating the Cytotoxic and Genotoxic Effects of Magnetic Iron Oxide Nanoparticles and Chitosan on Hepatocellular Carcinoma Cells (HCC)

**DOI:** 10.1007/s12013-024-01256-2

**Published:** 2024-04-01

**Authors:** Heba M. Fahmy, Samar Shekewy, Fathi A. Elhusseiny, Ahmed Elmekawy

**Affiliations:** 1 Biophysics Department, Faculty of Science, Cairo University, Cairo, Egypt; 2 Physics Department, Faculty of Science, Tanta University, Tanta, Egypt; 3 Physics Department, Faculty of Science, Menofia University, Menofia, Egypt

**Keywords:** Fe_3_O_4_ nanoparticles, Fe_3_O_4_-CS nanocomposite, HCC (HepG2), Comet assay, Oxidative and antioxidative stress markers

## Abstract

Hepatocellular carcinoma (HCC), the fifth most prevalent cancer worldwide, is influenced by a myriad of clinic-pathological factors, including viral infections and genetic abnormalities. This study delineates the synthesis, characterization, and the biological efficacy of iron oxide nanoparticles (Fe_3_O_4_) and chitosan-coated iron oxide nanoparticles (Fe_3_O_4_-CS) against HCC. Analytical methods confirmed the successful synthesis of both nanoparticles, with Fe_3_O_4_-CS demonstrating a smaller, uniform spherical morphology and distinct surface and magnetic properties attributable to its chitosan coating. The prepared materials were analyzed using various techniques, and their potential cytotoxic effects on HepG2 cancer cells line for HCC were investigated. In biological evaluations against HepG2 cells, a notable distinction in cytotoxicity was observed. Fe_3_O_4_ showed modest anticancer activity with an IC50 of 383.71 ± 23.9 µg/mL, whereas Fe_3_O_4_ exhibited a significantly enhanced cytotoxic effect, with a much lower IC50 of 39.15 ± 39.2 µg/mL. The Comet assay further evidenced Fe_3_O_4_-CS potent DNA damaging effect, showcasing its superior ability to induce apoptosis through extensive DNA fragmentation. Biochemical analyses integrated into our results reveal that Fe_3_O_4_-CS not only induces significant DNA damage but also markedly alters oxidative stress markers. Compared to control and Fe_3_O_4_-treated cells, Fe_3_O_4_-CS exposure significantly elevated levels of oxidative stress markers: superoxide dismutase (SOD) increased to 192.07 U/ml, catalase (CAT) decreased to 0.03 U/L, glutathione peroxidase (GPx) rose dramatically to 18.76 U/gT, and malondialdehyde (MDA) levels heightened to 30.33 nmol/gT. These results underscore the potential of Fe_3_O_4_-CS nanoparticles not only in inducing significant DNA damage conducive to cancer cell apoptosis but also in altering enzymatic activities and oxidative stress markers, suggesting a dual mechanism of action that may underpin their therapeutic advantage in cancer treatment. Our findings advocate for the further exploration of Fe_3_O_4_-CS nanoparticles in the development of anticancer drugs, emphasizing their capability to trigger oxidative stress and enhance antioxidant defense mechanisms.

## Introduction

Cancer stands as a formidable global health challenge, demanding effective strategies for prevention, early detection, and treatment. Hepatocellular carcinoma (HCC), ranking fifth globally in cancer incidence, exhibits various clinicopathological features, including genetic mutations and viral infections. Conventional cancer therapies like surgery, chemotherapy, and radiation, while effective, often entail non-specific side effects. Leveraging the targeted delivery capabilities of nanoparticles for anticancer drugs presents a promising avenue to enhance therapeutic efficacy and minimize damage to healthy tissues [1–7].

Magnetic nanoparticles (MNPs) have emerged at the forefront of cancer research, offering versatile applications in targeted drug delivery and magnetic hyperthermia [8]. Their capacity for functionalization with biomolecules allows for specific targeting of cancer cells, marking a significant advancement in drug delivery and imaging for cancer therapy. The utilization of external magnetic fields in magnetic targeting optimizes the precision of MNPs, demonstrating potential improvements in treatment outcomes. This approach addresses challenges associated with conventional therapies, such as low specificity, selectivity, and toxicity to healthy cells [9].

MNPs extend their impact to magnetic hyperthermia, wherein an alternating magnetic field induces heat to eliminate cancer cells effectively. This multifaceted application positions MNPs as a dynamic and effective alternative for cancer treatment. Ongoing research explores the use of natural compounds like tea polyphenols as chemo-preventive agents, capitalizing on their antioxidant and anticarcinogenic properties [10, 11].

The integration of MNPs into cancer therapy showcases significant potential for augmenting treatment effectiveness while minimizing toxicity. The emphasis on targeted drug delivery, magnetic hyperthermia, and the exploration of natural compounds highlights the continuous pursuit of innovative and efficient cancer therapies [12]. Oxidative stress, characterized by an imbalance in ROS production and the body’s detoxification capacity, is intricately linked to various diseases, including cancer [13]. The intricate interplay of antioxidant systems, such as glutathione and enzymes like SOD, CAT, and GPx, maintains intracellular redox balance, preventing mutations, cell death, and inflammation.

Antioxidants, exemplified by tea polyphenols, play a pivotal role in counteracting ROS-induced damage, restoring redox balance, and exhibiting direct anticancer properties. Enzymatic antioxidants like SOD, CAT, and GPx, alongside non-enzymatic counterparts like vitamins C and E, act as vital defenders against oxidative stress, crucial in averting damage to cellular structures [10, 14, 15].

The significance of antioxidants becomes more pronounced in diseases like hepatocellular carcinoma (HCC), a rapidly progressing form of liver cancer with limited effective treatment options. Superparamagnetic iron oxide nanoparticles (SPIONs), though promising in biomedical applications, raise concerns about DNA deterioration, oxidative stress, and micronuclei formation. Biochemical assays measuring oxidative stress markers, including SOD and glutathione, contribute valuable insights into the potential toxicity of SPIONs [16, 17].

Exposure to hazardous substances elevates ROS levels, and an inability to neutralize them leads to oxidative stress, damaging proteins, lipids, and DNA. Superparamagnetic iron oxide nanoparticles pose a risk, inducing oxidative stress and genetic material damage, emphasizing the need to comprehend their toxicity mechanism. Co-exposure with Chitosan-coated iron oxide MNPs showcases potential protective effects against oxidative damage induced by SPIONs [14].

Lipid peroxidation, initiated by ROS, contributes to cell membrane degradation and chronic diseases. The imbalance in antioxidant enzyme levels caused by SPIONs exacerbates lipid peroxidation, intensifying their adverse effects [17, 18].

Nanoparticles, a focus of extensive research, have diverse applications across consumer products, electronics, cosmetics, fabrics, and cleansing agents. They play a crucial role in biomedicine, particularly nanomedicine, improving drug delivery, diagnostics, and targeted treatment [7, 16, 19, 20]. Controlled shapes and sizes of nanoparticles make them ideal drug carriers, enhancing drug bioavailability and showing promise in cancer therapy, gene therapy, and vaccination, with potential improvements in efficacy and reduced side effects [15]. As nanoparticles become more prevalent, comprehensive research on their toxicity, termed nanotoxicology, becomes increasingly crucial [21].

Iron oxide nanoparticles, known for their magnetic properties, are extensively applied in biomedical and environmental domains due to their small size, biocompatibility, and superior magnetism [14]. These nanocrystals exhibit unique physical and chemical properties at the nanoscale, making them ideal for various applications in nanoscience and nanotechnology. Biocompatible and cost-effective, iron oxide nanoparticles are valuable tools in research and development, proving beneficial for targeted molecule delivery in biomedical applications such as MRI contrast agents, drug delivery, and cancer therapy [22, 23].

However, modified MNPs, including iron oxide, face challenges like high aggregation, impacting their effectiveness in biomedical applications. Strategies like surface chemistry modification and coating with organic or inorganic materials, such as polymers, lipids, proteins, silica, alumina, or titanium dioxide, are employed to enhance stability, biocompatibility, and functionalities [24, 25]. The choice of coating material is critical, influencing surface charge, surface energy, and interactions between particles, and is essential for obtaining nanoparticles with the desired stability and functionality [9, 26].

CS, an acetylated derivative of chitin, exhibits biocompatibility, bioactivity, and biodegradability, making it easily functionalized for biomedical applications [27]. CS is widely recognized as a body-friendly material used in creating drug forms such as gels, films, and nanoparticles. Notably, to address the common challenge of high aggregation in MNPs, surface engineering techniques are employed. This involves applying various stabilizer coatings, including surfactants, synthetic polymers, and natural polymers, to enhance the stability and biocompatibility of the nanoparticles. When CS is used to make nanoparticles, they typically acquire a positive charge and exhibit strong adhesion to mucus membranes. This unique property not only enhances biocompatibility but also facilitates the controlled release of drugs. The positive charge assists in their interaction with biological membranes, while the adhesion property ensures sustained drug release, enabling effective transport through cells. Moreover, the inherent properties of pure CS contribute to its biocompatibility and anti-cancer potential. Studies have demonstrated that CS itself possesses anti-cancer properties against various types of cancer [28–32].

These coatings reduce the surface energy of MNPs, preventing clumping and potentially altering contrast imaging properties for applications like magnetic resonance imaging [33]. Selection of coating materials depends on the desired properties and application. Polyethylene glycol (PEG) is commonly used for reducing toxicity and increasing stability, while Chitosan enhances biocompatibility and targeting efficiency of MNPs [14]. Chitosan-coated iron oxide nanoparticles, prepared using ultrasonic methods with a 1% Chitosan solution in 0.5% acetic acid, demonstrate reduced toxicity and increased stability [27, 34]. Studies indicate that a small coating density of 5 mg Chitosan per 1 g of magnetite significantly decreases toxicity [14]. In a previous study, investigations revealed that both Fe_3_O_4_ and NiFe_2_O_4_ nanoparticle samples exhibited no cytotoxicity towards four different cell types prior to chitosan coating. Interestingly, after chitosan coating, the Fe_3_O_4_-CS nanocomposite demonstrated heightened growth inhibitory activity against human hepatocellular carcinoma HepG2, with an IC50 value of 74.60 ± 8.10 µg/ml [3].

The aim of this study lies in the exploration of cytotoxic and genotoxic effects exerted by magnetic iron oxide nanoparticles (IONPs) and Chitosan-coated IONPs (CS-IONPs) specifically on hepatocellular carcinoma cells (HCC). The unique approach involves the synthesis of pure Fe_3_O_4_ nanoparticles and their effective coating with Chitosan (CS), a natural biopolymer, to enhance biocompatibility and enable precise targeting of cancer cells. This investigation contributes to the understanding of the potential therapeutic applications of these nanomaterials in the context of hepatocellular carcinoma.

## Materials and Methods

### Synthesis of Magnetic Fe_3_O_4_ Nanoparticles

FeCl_3_·6H_2_O and FeCl_2_·4H_2_O in a ratio of 1.62:0.99 grams were dissolved in 40 ml of deionized water. The magnetic Fe_3_O_4_ nanoparticles were synthesized using the co-precipitation method. Subsequently, 5 ml of ammonia solution (28% w/v%) was introduced, followed by the addition of 4.4 grams of sodium citrate after a 10-minute interval. The reaction temperature was elevated to 90 °C with continuous stirring for 30 min. After cooling, the residue underwent two rounds of rinsing with acetone to eliminate excess free citrate. Throughout the rinsing process, the sample was separated from the supernatant using a permanent magnet. Finally, the sample was subjected to drying in a vacuum pump without the application of heat.

### Synthesis of Chitosan-coated Magnetic Fe_3_O_4_ Nanoparticles

The surface of magnetic Fe_3_O_4_ nanoparticles underwent modification through a coating process with a chitosan (CS) solution. In this procedure, 0.25 g of magnetic Fe_3_O_4_ was dispersed in a surfactant-containing cetyl trimethyl ammonium bromide (CTAB) solution (2 grams of CTAB dissolved in 400 ml of deionized water), denoted as “solution c”. Subsequently, 100 ml of chitosan solution (0.02 grams of CS powders dissolved in 100 ml of a 1% (w/v) acetic acid solution) was slowly added dropwise into solution c. The resulting mixture underwent continuous stirring at a rotational speed of 1000 rpm for one hour at room temperature.

Following the stirring period, a magnet bar was employed to magnetically separate chitosan-coated MNPs (CS) from the solution. The separated particles were thoroughly washed with ethanol and deionized water multiple times. Finally, the obtained nanoparticles were dried overnight at 60 °C.

## Physical and Chemical Characterization of the Prepared Formulations

### Ultraviolet-visible (UV-Vis) Spectroscopy

The automated UV-vis Perkin-Elmer Model Lambda 20 spectrophotometer was used to perform the spectroscopic measurements in the range from 200 to 700 nm in solution form.

### Fourier-transform Infrared (FTIR) Spectroscopy

Infrared spectra were recorded by a PerkinElmer 1650 Fourier transform infrared spectroscopy (4000–400 cm^−1^) by KBr disks. The device was continuously purged with dry air to remove the water vapor from the atmosphere. Via this apparatus, apparent changes in the structure of the compound can be observed by studying the variations of bandwidth and frequency of the vibration modes.

### Transmission Electron Microscopy (TEM)

The morphology and structure of Fe_3_O_4_ nanoparticles and CS-Fe_3_O_4_ nanoparticles were analyzed using TEM imaging (JEM 1230 electron microscope, Jeol, Tokyo, Japan). A small amount of diluted sample was applied to a copper-coated carbon grid and allowed to air-dry for approximately 15 min. Excess dispersion was removed using filter paper, and the grid was left to air-dry further. Subsequently, the samples were examined using a high-resolution transmission electron microscope.

### Dynamic Light Scattering (DLS)

Dynamic light scattering (DLS) analysis was conducted using the Zetasizer Nano ZS90 (Malvern Instruments, UK) to determine the particle size of Fe_3_O_4_ nanoparticles and CS-Fe_3_O_4_ nanoparticles at 25 °C. Samples were appropriately diluted with deionized water before analysis. The mean and distribution of the hydrodynamic diameter of the two samples were then determined. The instrument was equipped with a He–Ne laser radiation beam at 633 nm, and the sample suspension was placed in the sample holder. Back-scattered light was detected by a photomultiplier tube, and the average size of the samples was calculated.

### Zeta Potential (ZP)

DLS not only provides particle size information but also offers insights into surface charges, known as Zeta Potential. The Zeta Potential was measured using the same capillary cuvette immediately after determining the particle size, utilizing the Zetasizer Nano ZS90 from Malvern Instruments, UK. The magnitude of the zeta potential holds significance in understanding the physical stability of nanoparticles.

### Vibrating Sample Magnetometer (VSM)

VSM analysis in this study is crucial for investigating the magnetic properties of the synthesized nanoparticles. MNPs hold significant importance in the development of localized drug delivery systems for cancer patients. These nanoparticles serve as carriers for drugs, specifically targeted to tumors. Once the drug-loaded nanoparticles enter the patient’s bloodstream, the application of a magnetic field helps retain these particles precisely at the tumor site. This innovative approach represents a promising strategy in cancer treatment, offering potential advancements in targeted and effective therapeutic options for patients [35].

A magnetic hysteresis curve is obtained by vibrating the sample magnetometer. The applied magnetics field was changed, and magnetization properties of synthesized Fe_3_O_4_ and chitosan-coated Fe_3_O_4_ nanoparticles were measured at 37 °C.

## Biological Studies

### Cell Lines

HepG2, human liver carcinoma cell lines were provided from ATCC (The American Type Culture Collection; Manassas, VA, USA) thru VACSERA (The holding company for biological products and vaccines; Giza, Egypt).

### MTT Assay (Cell Viability)

Using the MTT assay technique, Fe_3_O_4_ nanoparticles and Fe_3_O_4_-CS were tested for their anticancer potential in HepG2 cells during a 48-hour period. Based on metabolic activity, the colorimetric and sensitive MTT test measures cell viability quantitatively. The assay is based on the reduction of 3-(4,5-dimethylthiazol-2-yl)-2,5 diphenyl tetrazolium bromide (MTT), a yellow, water-soluble substrate. This reduction is mainly catalyzed by the enzyme mitochondrial lactate dehydrogenase (LDH) in live cells. Through an enzymatic process, MTT is transformed into insoluble crystals of (E, Z)-5-(4,5-dimethylthiazol-2-yl)-1,3-diphenyl formazan (Formosan), which have a purple or dark blue color. When the insoluble formazan is dissolved in DMSO, a black color is produced that is closely correlated with the quantity of cells and a measure of cell viability. Preceding treatment, HepG2 cell lines were precultured and then seeded in a 96-well microtiter plate (104 cells/well) for 24 h, allowing cells to attach to the plate wall. The investigated substances were added to the cells at different doses (from 4.88 to 10,000 µg/ml), and match wells were made for each dosage. The monolayer cells were cultured for 48 h at 37 °C with 5% CO_2_ in the air. After four hours of exposure, the media were taken out and 40 µl of MTT solution was applied to each well. Next, 200 µl of DMSO was added to each well, and the mixture was gently shaken for 10 min at room temperature to solubilize the MTT crystals. A microplate reader was used to measure absorbance at 570 nm. Following the designated incubation period, the survival curve for each cell line was created by graphing the relationship between the drug concentration and the surviving fraction [36–38] The concentration at which 50% inhibition of cell viability (IC50) occurred was computed.

To establish a baseline for cell viability, the cells were cultured in the culture media alone (untreated wells). The treated cells were inspected under a microscope to check for morphological alterations and separated cells [37, 38].

Moreover, optical densities (OD) were measured using a Biotek 8000 ELISA plate reader, and the USA (Unit of Specific Absorbance) and half-maximal inhibitory dosages (IC50) were calculated using the Masterplex-2010 application. The findings came from three different trials [39]. The proportion of viable cells is equivalent to (treated cell optical density / untreated cell optical density) times 100%.

### HepG2 Cells Treatment with their IC50 of Compounds

HepG2 cells were grown in a 75 cm^2^ flask and treated with the indicated IC50 dosages in order to measure genotoxicity and analyse oxidative stress indicators generated by exposure to their respective IC50 concentrations, suggestive of a 50% reduction in cancer cell growth. Following treatment, the cell culture was centrifuged for 15 min at 4000 rpm at 2–8 °C. The comet test was used to assess the genotoxicity of the resultant pellet, and oxidative stress parameters were measured by isolating the supernatant that included cellular components. For further examination, the supernatant was quickly frozen at −70 °C. Every evaluation pertaining to indicators of oxidative stress and genotoxicity was conducted at the Animal Reproduction Research Institute located in Giza, Egypt.

### Evaluation of DNA Damage using the Comet Assay

The objective of the straightforward genotoxicity procedure known as the comet test, or single-cell gel electrophoresis, is to assess cell DNA strand breakage [3]. Comet percentage, tail length, percentage of DNA in the tail, and olive moment were significant metrics utilized to assess DNA damage. Using agarose as a soaking medium, the cells were lysed on a microscope slide with a high-salinity solution and detergent to produce nucleoids, which are composed of supercoiled DNA loops connected to the nuclear matrix. The slide was removed from the lysis solution and allowed to sit at room temperature in an alkaline solution for forty minutes while the outside world was dark.

Subsequently, the slide was left to air dry for twenty minutes. This was followed by two five-minute Tris-Borate EDTA (TBE) washes, and low-voltage electrophoresis. SYBR green, which is specifically designed for the comet assay, was applied to the slide, and it was then allowed to air-dry for six hours at room temperature. The stained slide was examined under a fluorescence microscope, and the data were analyzed using the LAI Comet test analysis software.

### Identification of Markers for Oxidative Stress and Antioxidative Activities

The activity levels of GPx, CAT, and SOD were measured, together with MDA levels, in order to assess potential material toxicity in HepG2 cells.

### Measuring the Activity of Superoxide Dismutase (SOD)

Because they aid in the conversion of the superoxide anion into molecular oxygen and hydrogen peroxide, SOD are essential components of the cellular antioxidant defense system. The measurement of SOD activity depends on the enzyme capacity to block the reduction of Nitroblue tetrazolium dye (NBT) in the presence of NADH, which is mediated by phenazine methosulphate (PMS). For five minutes at 25 °C, the sample’s increased absorbance was seen at a wavelength of 560 nm [12]. The SOD activity was determined in units per milliliter sample (U/ml).

### Assessment of Catalase Activity using the CAT Assay

An essential part of the body’s defense against hydrogen peroxide (H_2_O_2_), a strong oxidant that can cause intracellular damage, is the antioxidant enzyme CAT, which is present in the majority of aerobic cells. An essential technique for examining oxidative stress is the CAT assay, which has been included in the repertory of antioxidant biomarkers. CAT reaction with a certain amount of H_2_O_2_ was used to evaluate CAT activity after the Aebi technique [40].

At 25 °C, the CAT inhibitor precisely stopped the process after 1 minute. Any leftover H_2_O_2_ in the presence of peroxidase reacted with 4-aminophenazone (AAP) and 3,5-Dichloro-2-hydroxybenzene sulfonic (DHBS) acid to form Quinoneimine Red Dye, a chromophore whose color intensity was inversely proportional to the CAT concentration in the samples. Following a 10 min incubation period at 37 °C, the absorbance of the samples was measured at 510 nm, and the CAT activity was reported in units per liter (U/L).

### Measuring the Activity of Glutathione Peroxidase (GPX)

Within the GPx enzyme family, cellular glutathione peroxidase (c-GPx) is essential for the detoxification of peroxides in cells. Since peroxides can break down into extremely reactive radicals, the GPx enzyme serves as a defense mechanism, protecting the cell from the damaging effects of free radicals, especially lipid peroxidation. This assay uses an indirect approach to measure the activity of c-GPx. This technique uses a UV spectrophotometric approach and involves adding glutathione reductase, β-nicotinamide-adenine dinucleotide phosphate reduced (NADPH), and glutathione (GSH) to the sample. The enzyme reaction starts as soon as hydrogen peroxide (H_2_O_2_) is added to the mixture. Subsequently, the reduction in absorbance is measured at 340 nm/min for three minutes. The rate of decrease is directly proportional to the GPx activity present in the sample, and the results are expressed in U/g tissue.

### Quantification of Lipid Peroxidation Levels using the MDA Assay

MDA is a byproduct of peroxidation in cells, particularly resulting from the peroxidation of polyunsaturated fatty acids. Elevated levels of free radicals can instigate lipid peroxidation, leading to an excessive production of MDA. Measurement of MDA levels serves as an indicative marker for lipid peroxidation, offering valuable insights into oxidative stress conditions [41]. Thio barbituric acid (TBA) and MDA react in an acidic media for 30 min at 95 °C to produce the Thio barbituric acid reactive product, which is used to measure MDA levels. At 534 nm, the absorbance of the resulting pink product is determined [42]. MDA levels are quantified in terms of nmol/ml.in terms of nmol/ml.

### Statistical Analysis

The data generated in this study were derived from a minimum of three independent experiments, and the outcomes were expressed as the mean ± standard deviation (SD). Statistical analyses, including comparisons for both independent and dependent materials, were performed using SPSS version 26, with significance considered at *P* < 0.05.

## Results and Discussion

### FTIR Spectroscopy Analysis

**Fig. 1:**
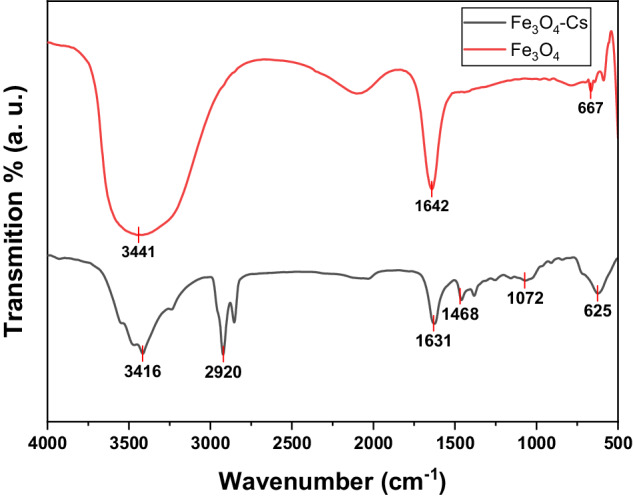
FTIR spectrum of Fe_3_O_4_ nanoparticles and Fe_3_O_4_-CS

Figure 1 illustrates the FTIR spectra of the synthesized Fe_3_O_4_ nanoparticles and Fe_3_O_4_-CS within the temperature range of 4000–400 cm^−1^ at room temperature. The FTIR analysis was conducted to validate the formation of the spinel structure and the synthesis of Fe_3_O_4_ and Fe_3_O_4_-CS nanomaterials. A broad band observed around 3400 cm^−1^ corresponds to the O˗H stretching vibration, indicating the OH group stretching vibration mode within the 3200–3600 cm^−1^ range. For the pure Fe_3_O_4_ nanoparticles, the spectrum exhibited three distinctive peaks at 667, 1642, and 3441 cm^−1^, representing the stretching and torsional vibration modes of Fe–O bonds in the tetrahedral and octahedral sites, respectively. The FTIR spectrum of Fe_3_O_4_-CS nanomaterials displayed characteristic peaks associated with both CS and iron oxide. The robust absorption peak at 625 cm^−1^ confirmed the presence of the Fe–O bond core in the Fe_3_O_4_ nanoparticles. Simultaneously, absorption peaks related to CS were observed at 1072, 1468, 1631, 2920, and 3416 cm^−1^, indicating the successful coating of Fe_3_O_4_ nanoparticles by the CS.

shifts in the NH bending vibration band (from 1642 to 1631 cm^−1^) and the Fe–O stretching band (from 667 to 625 cm^−1^) were seen upon coating with CS, suggesting the attachment of iron ions to the NH2 group of CS. Furthermore, it was suggested that the surface-charged Fe_3_O_4_ nanoparticles interacted with the cationic biopolymer matrix of CS through electrostatic interactions and hydrogen bonding with the –NH_2_/OH group, forming a hybrid nanomaterial, based on the shifts in the O–H and N–H stretching vibrations (from 3441 to 3416 cm^−1^) [3].

### TEM Analysis

**Fig. 2:**
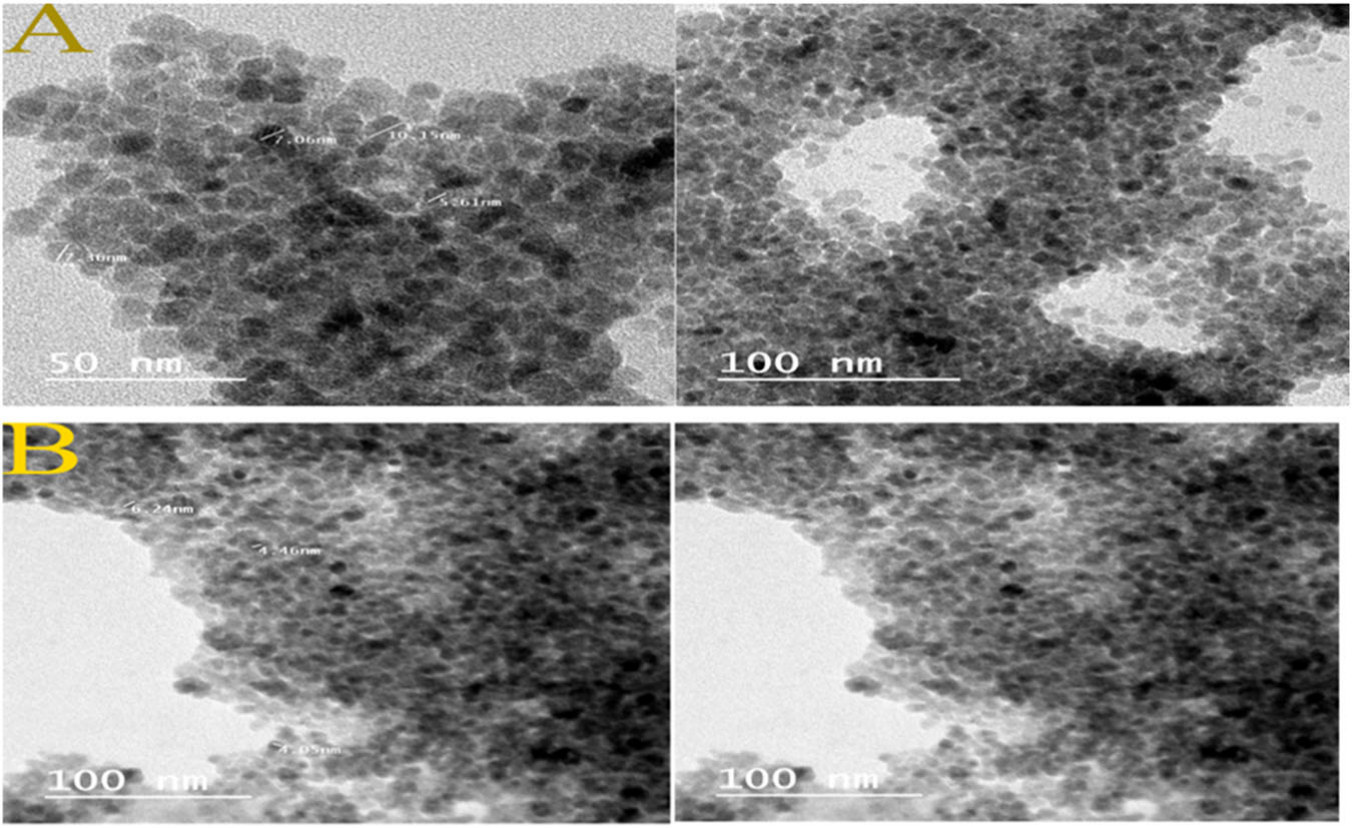
TEM images of (**A**) Fe_3_O_4_ nanoparticles and (**B**) Fe_3_O_4_-CS

**Fig. 3:**
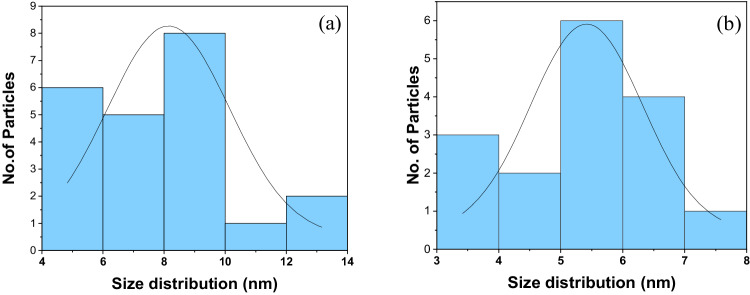
Size distribution of (**a**) Fe_3_O_4_ nanoparticles and (**b**) Fe_3_O_4_-CS

**Fig. 4:**
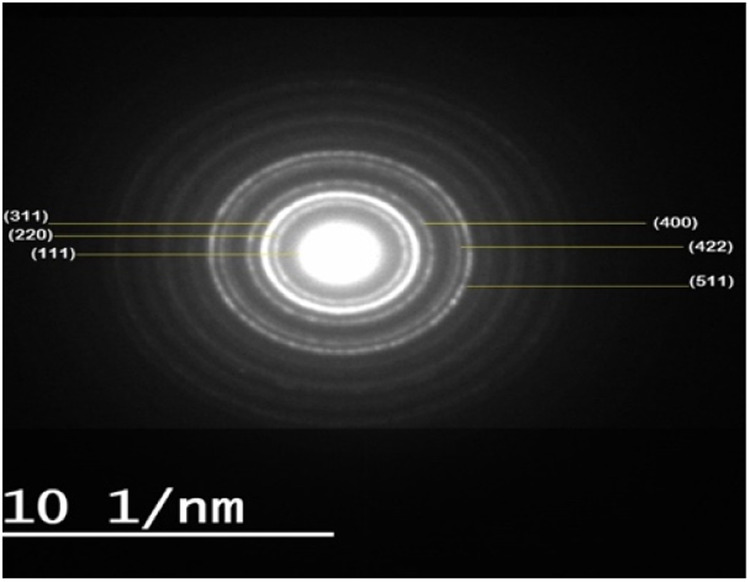
The selected area electron diffraction pattern of (Fe_3_O_4_)

TEM has observed the size and morphology of synthesized Fe_3_O_4_ and Fe_3_O_4_-CS. Images obtained demonstrated that, in comparison to Fe_3_O_4_, the synthesized Fe_3_O_4_-CS are almost spherical and have a more uniform size distribution. The average diameters are around 6.03 ± 1.89 nm (bare MNP) and 4.92 ± 1.17 nm (CS MNP) and shows in Fig. 2. The histogram (Fig. 3) shows the particle size distribution, the size of Fe_3_O_4_ particles varies from 4–14 nm with an average size of 7.9 nm. The size of Fe_3_O_4_-CS particles varies from 3–8 nm with an average size of 5.4 nm. The selected area electron diffraction pattern (Fig. 4) shows the different interplanar spacing of (111), (220), (311), (400), (422) and (511) with diffracting conditions and matched well with the XRD pattern, which also confirms the structure of the prepared materials.

### Dynamic light scattering (DLS)

**Fig. 5:**
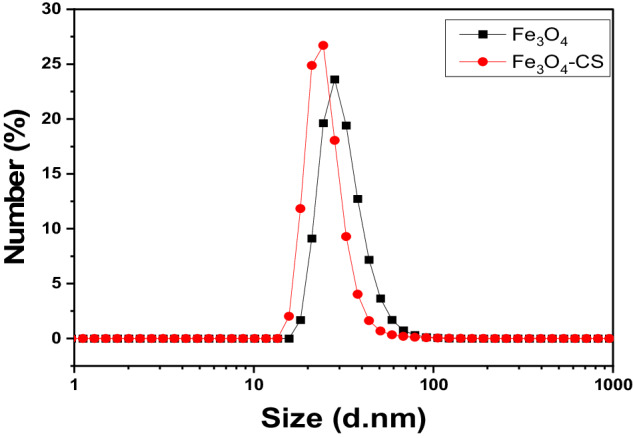
Particle Size Distribution of Fe_3_O_4_ nanoparticles and Fe_3_O_4_-CS

The particle size analysis was performed using dynamic light scattering technique. The average hydrodynamic size for Fe_3_O_4_ nanoparticles and Fe_3_O_4_-CS were 31.80 ± 9.90 nm and 25.59 ± 8.14 nm, respectively (see Fig. 5). The difference in the size found via the DLS technique compared to that found via TEM could be returned to that DLS measures the hydrodynamic diameter, which means that the primary particle size plus the layer of hydration around nanoparticles in the aqueous medium [43].

### Zeta Potential (ZP) Analysis

**Fig. 6:**
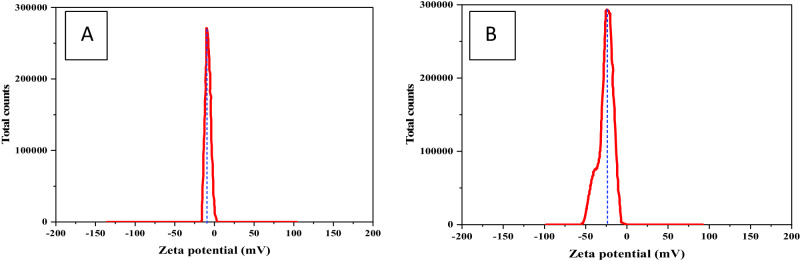
Zeta Potential distribution of (**A**) Fe_3_O_4_ nanoparticles and (**B**) CS- Fe_3_O_4_

ZP serves as a valuable characterization technique for determining the surface charge of nanoparticles in solutions, a crucial aspect in the development of drug delivery carriers. When charged particles exist in a solution, their surfaces attract molecules with opposite charges, forming a thin liquid layer known as the stern layer. As the particle diffuses, an outer layer with loosely associated ions surrounds it. The term of ZP refers to the electrical potential of this resulting double layer. Nanoparticle zeta potentials typically range between −100 and +100 mV, with an increasing zeta value signifying greater stability and categorizing them as strongly cationic or strongly anionic [44]. Figure 6 illustrates the mean zeta potential for the examined Fe_3_O_4_ nanoparticles and Fe_3_O_4_-CS as −8.30 ± 3.55 mV and −25 ± 4.88 mV, respectively. The increase in mean zeta potential for Fe_3_O_4_-CS indicates enhanced stability due to the negative surface charge resulting from the hydrolysis of surface metal ions. Given that most cellular membranes carry a negative charge, the zeta potential plays a role in a nanoparticle permeation tendency. The negative value of Fe_3_O_4_-CS signifies high stability and homogeneity attributed to the repulsive forces among particles [45, 46].

The calculated Polydispersity Index (PDI) values for the Fe_3_O_4_ and Fe_3_O_4_-CS nanoparticles were found to be 0.361 and 0.417, respectively. These figures signify a moderately uniform size distribution for both nanoparticle types, with the slightly higher PDI for Fe_3_O_4_-CS attributed to the chitosan coating process. The inherent variability introduced by the non-uniform thickness of the chitosan coating around different nanoparticles highlights the intricate dynamics involved in the coating process, influencing the ultimate size and distribution of the coated nanoparticles. The significance of these PDI values becomes particularly pronounced in the context of biological applications. A moderate level of polydispersity (PDI < 0.5) indicates that the nanoparticles possess sufficient uniformity for a wide range of biomedical applications.

### UV-Vis Spectroscopy Analysis

**Fig. 7:**
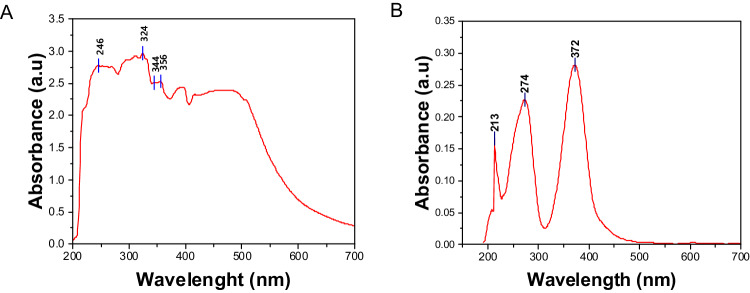
The UV-vis absorption spectrum of the prepared (**A**) Fe_3_O_4_ nanoparticles and (**B**) Fe_3_O_4_-CS

UV-vis spectroscopy is a crucial tool for confirming the successful synthesis of nanoparticles and was employed to monitor the formation of nanomaterials. The spectral analysis covered a range of 200–700 nm, as illustrated in Fig. 7. The synthesized Fe_3_O_4_ exhibited a distinct spectral absorbance peak at 324 nm, a characteristic feature associated with Fe_3_O_4_ in the 300–400 nm range. Following the addition of a chitosan solution to the synthesized Fe_3_O_4_, a notable shift in the maximum absorbance was observed at 372 nm. Moreover, the absorption wavelength in the 260–300 nm range in Fe_3_O_4_ corresponds to chitosan, indicating π → π* transitions of C = O [47]. The observed changes in the absorbance spectrum, characterized by broadening, provide initial evidence supporting surface modifications of Fe_3_O_4_ [48].

### Vibrating Sample Magnetometer (VSM) Analysis

**Fig. 8:**
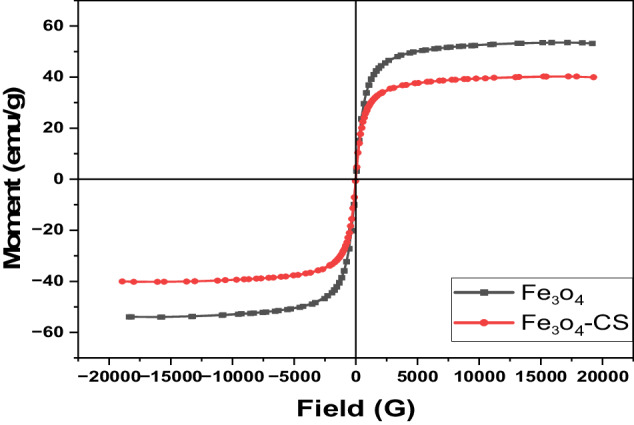
The Magnetic hysteresis curves of the prepared Fe_3_O_4_ nanoparticles and Fe_3_O_4_-CS

A magnetic hysteresis curve was generated through the operation of a vibrating sample magnetometer, involving a variation in the applied magnetic field to measure the magnetization properties of the synthesized Fe_3_O_4_ and Chitosan-coated Fe_3_O_4_ nanoparticles at 37 °C. The magnetic hysteresis curves (Fig. 8) revealed that the saturation magnetization (Ms) of Fe_3_O_4_ was 53.56 emu/g, whereas for Chitosan-coated Fe_3_O_4_, it was 40.24 emu/g. This is consistent with the idea that, under the assumption that ionic configurations remain unchanged, Ms values in nanostructured materials are often less than those in bulk materials. Significantly, the Ms value of Fe_3_O_4_-CS revealed a thin coating of chitosan on the nanoparticles, which makes it difficult to detect the magnetization statistically. This result is in line with TEM pictures. The magnetic hysteresis curves clearly show that these nanoparticles behave superparamagnetically, which is supported by the absence of remanence or coercivity in the magnetization curves and the nanosize of the particles. On the other hand, superparamagnetic nanoparticles are useful for a number of biological uses, such as cancer treatment for hyperthermia and as magnetic resonance imaging contrast agents [49].

## Biological Studies

### Impact of Nano Formulations on HepG2 Cells - Cytotoxicity Assessment

**Fig. 9:**
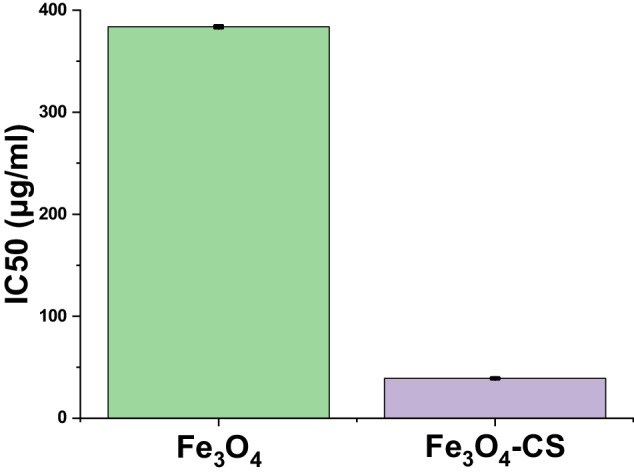
Cytotoxic activity (IC50 in μg/ml) for two nanomaterials materials against the HepG2 cell line

**Fig. 10:**
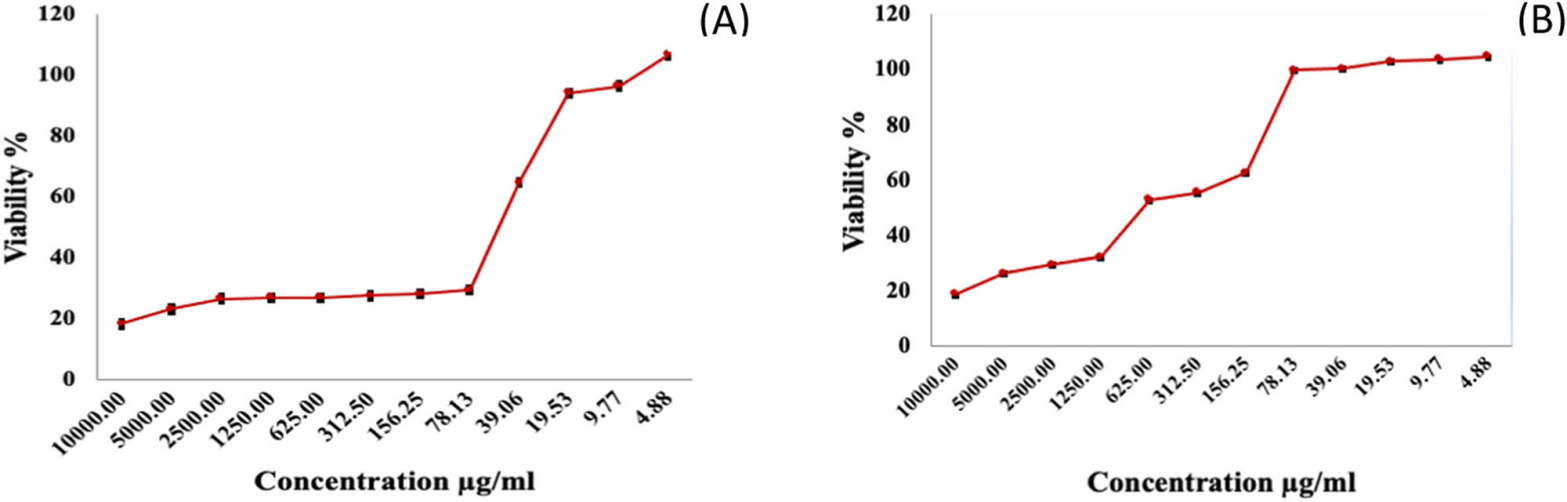
Effect of (**A**) Fe_3_O_4_ and (**B**) Fe_3_O_4_-CS on cell viability of HepG2 cancer cells. The results presented in this study are based on at least three separate experiments, with values shown as the average ± standard deviation (SD)

The MTT assay was conducted on HepG2 cells line to assess the antiproliferative effects of the both materials (refer to Fig. 9), spanning a cell incubation period of 48 h. The relation between relative cell viability and medication concentration was graphically represented to generate the survival curve of HepG2 cells (see Fig. 10), wherein the corresponding IC50 values are indicated. IC50 represents the concentration capable of diminishing the proliferation of cancer cells.

The in vitro cytotoxicity study of the compounds against HepG2 cells revealed that Fe_3_O_4_ demonstrated weak anticancer activity, exhibiting an IC50 value of (383.71 ± 23.9 μg/ml) against this specific cancer cell line. In contrast, Fe_3_O_4_-CS exhibited significant activity with IC50 concentrations of 39.15 ± 39.2 μg/ml, showcasing a highly potent cytotoxic impact on HepG2 cancer cells. The IC50 values underscore the notable cytotoxic efficacy of Fe_3_O_4_-CS. This can lead to this substance being classed as a chemotherapeutic agent.

### Evaluation of DNA Damage using Comet Assay

**Fig. 11:**
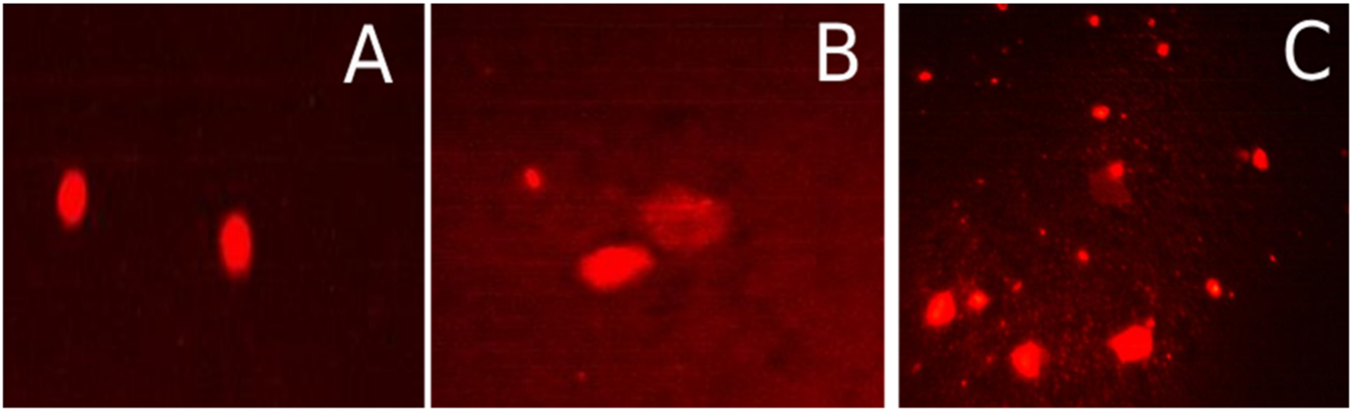
Fluorescence-stained comet images of HepG2 cells. **A** Control cells (untreated cells), (**B**) Treated cells with Fe_3_O_4_, (**C**) Treated cells with Fe_3_O_4_-CSE

The Comet assay revealed the photo-induced DNA damaging capacity of the compounds at the individual cell level, commonly employed to distinguish single- and double-strand breaks as genotoxic features. HepG2 cells, in conjunction with untreated control cells, underwent exposure to the respective IC50 concentrations of the compounds for 48 h. The DNA of untreated control cells showed no comet formation, indicating intact DNA. Results were presented through fluorescence-stained comet images (Fig. 11) and covered five critical parameters: percentage of damage (comet%), % DNA in the tail, tail length, and olive moment. This comprehensive assessment offered insights into DNA damage. Tail length signifies the extent of DNA migration from the nuclear core to the anode under electrical flux, while % DNA in the tail indicates the proportion of damaged DNA. The olive moment is particularly effective in identifying heterogeneity within a cell population by detecting variations in DNA distribution within the tail. DNA damage is quantified by measuring the distance between the nucleus genetic material (comet head) and the subsequent ‘tail.’

The analysis of DNA damage through the Comet assay unveiled distinct parameters among various samples, including the control, Fe_3_O_4_, and Fe_3_O_4_-CS. The Comet %, reflecting DNA fragmentation, registered at 10.2% for the control, denoting baseline damage levels. Conversely, Fe_3_O_4_ exhibited an elevated Comet % of 14.6%, implying a heightened degree of DNA fragmentation. Remarkably, the Fe_3_O_4_-CS sample demonstrated a further increase in Comet %, reaching 16.5%, suggesting a potentially more significant impact on DNA integrity. Tail length, indicating DNA migration extent, measured at 6.9 px for the control, 8.9 px for Fe3O4, and 7.7 px for Fe_3_O_4_-CS. % DNA in Tail, representing the proportion of damaged DNA, escalated from 7.9% (control) to 8.2% (Fe_3_O_4_) and significantly to 15.5% (Fe_3_O_4_-CS). Tail moment and Olive tail moment, reflecting DNA damage intensity, exhibited similar trends, with Fe_3_O_4_-CS displaying the highest values. These observations imply that Fe_3_O_4_-CS may induce more pronounced DNA damage compared to both the control and Fe_3_O_4_ samples.

**Fig. 12:**
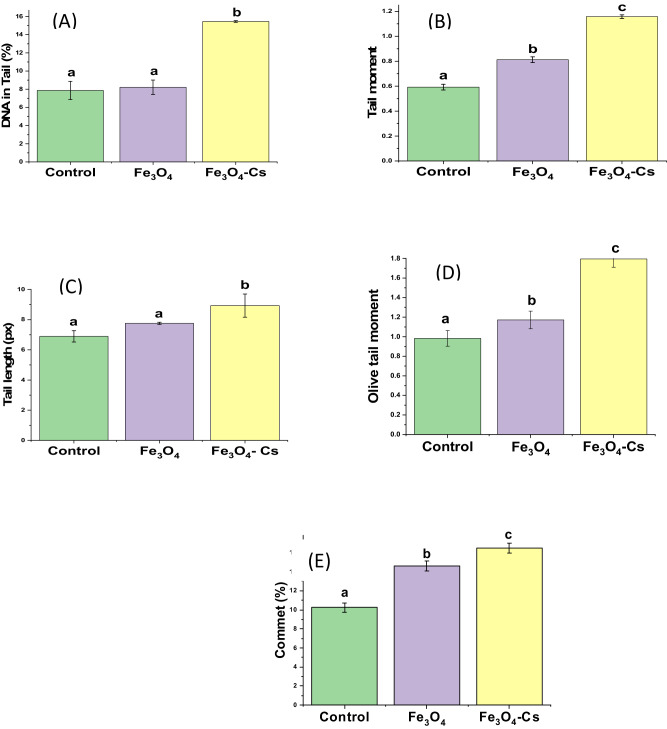
The comet assay assessed the DNA damage of HepG2 cells (**A**) Tail moment, (**B**) Tail length, (**C**) DNA in Tail %, (**D**) Olive Tail moment and (**E**) Commet %. Different letter (a, b, c) means a significant difference between control, Fe_3_O_4_ and Fe_3_O_4_-CS nanoparticles (at *p*-value < 0.05), also the same letters indicate non-significant changes

Figure 12 displays the mean values of all parameters for cells subjected to treatment with Fe_3_O_4_, Fe_3_O_4_-CS, and the control group. When compared to cells treated with Fe_3_O_4_, Fe_3_O_4_-CS showed a highly significant effect on DNA damage and indicated a faster migration of DNA to the anode. The findings of the comet test showed that HepG2 cells DNA damage increased with material concentration, with Fe_3_O_4_-CS showing a very low IC50 value and offering a therapeutic benefit in the treatment of cancer. Consequently, Fe_3_O_4_-CS demonstrated the potential to induce DNA fragmentation, leading to apoptosis and triggering genetic material breakdown, including DNA double-strand breaks.

### Determination of Oxidative Stress Markers

After 48 h of HepG2 exposure to the IC50 concentrations of the materials, it is imperative to scrutinize the effects of Fe_3_O_4_ and Fe_3_O_4_-CS on antioxidant defenses such as SOD, CAT, GPx, and the oxidant MDA. Cancer cells, driven by oncogenic activation, heightened metabolic activity, and mitochondrial damage, generate various ROS [21], High levels of oxidative stress, characterized by an increased production of ROS, can potentially induce cytotoxicity, impede cell growth, and initiate cell death through apoptosis/necrosis. The role of antioxidants in mitigating ROS is crucial. Endogenous antioxidative enzymes such as SOD, CAT, and GPx play a pivotal role in managing cellular oxidative stress [50]. Extended periods of oxidative stress cause a change in antioxidant activity. SOD are metalloenzymes that catalyse the conversion of superoxide anions into molecular oxygen and hydrogen peroxide, which is an essential component of cells’ antioxidant defense system [51].

**Fig. 13:**
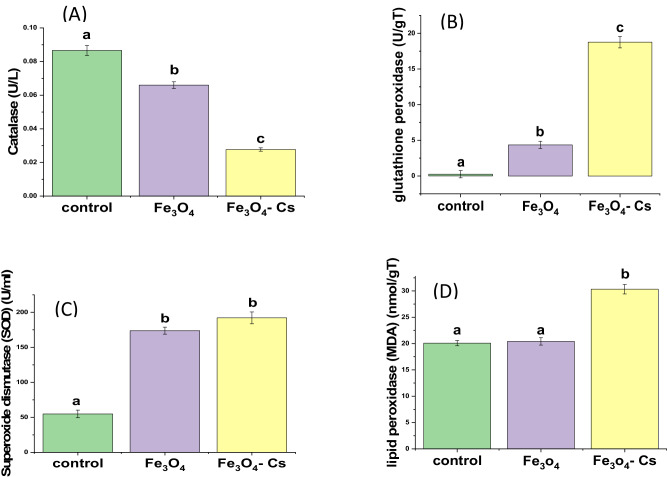
The activity of antioxidants (**A**) CAT, (**B**) GPx, and (**C**) SOD and the oxidant (**D**) MDA levels in HepG2 after 48 h incubation with Fe_3_O_4_ and Fe_3_O_4_-CS. Different characters indicate significant (*p* < 0.05) different mean. Different letter (a, b, c) means a significant difference between control and Fe_3_O_4_, Fe_3_O_4_-CS nanoparticles (at *p*-value < 0.05), also the same letters indicate non-significant changes

After treating HepG2 cells with Fe3O4 and Fe_3_O_4_-CS, as depicted in Fig. 13, the synthesized materials exhibited a notable increase in SOD activity. Cancer cells tend to generate higher levels of ROS compared to healthy cells, playing a role in cancer initiation and progression [52]. It has also been observed that the antioxidant levels generally elevate in various types of cancer. SOD, being a ubiquitous antioxidant with low levels in cancer cells, might experience an upsurge in activity due to the damage induced by heightened ROS levels [53]. In the current study, the elevated SOD activity suggests that, initially, as a response to cancer, ROS levels are elevated, and SOD increases to counteract the heightened ROS levels.

Moreover, heightened SOD activity triggers increased production of ROS in the HepG2 cells under examination, subsequently inducing cell apoptosis. The majority of aerobic cells have the vital antioxidative enzyme CAT, which is involved in the body’s defense against hydrogen peroxide (H_2_O_2_), a strong oxidant that can cause intracellular damage. Following the treatment with Fe_3_O_4_ and Fe_3_O_4_-CS, a decrease in CAT activity is observed, possibly attributed to the damage induced by ROS. Nevertheless, the CAT enzyme serves an anti-apoptotic and protective function by eliminating ROS, and its suppression may elevate ROS levels, leading to oxidative damage [54]. This suggests that the CAT role in converting H_2_O_2_ into H_2_O and O_2_ might be compromised in cancer, impairing its antioxidant action. The observed higher SOD activity also correlates with a reduced production of H_2_O_2_.

In the current study, it was observed that Fe_3_O_4_-CS led to a significant increase in GPx activity. These findings underscore the enhanced antioxidant capacity of the antioxidant system in response to oxidative stress, highlighting the crucial role of the GPx enzyme in detecting oxidative stress sensitivity. Figure 13 illustrates the GPx activity in both control and treated HepG2 cells, revealing a notable increase compared to the control cells. c-GPx belonging to the GPx enzyme family, plays a pivotal role in detoxifying peroxides within cells. Given that peroxides can decompose into highly reactive radicals, the GPx enzyme is instrumental in safeguarding cells against free radical damage, particularly lipid peroxidation [55]. The observed correlation between GPx levels and the incidence of ROS indicates the cell’s reliance on GPx for detoxifying free radicals. Furthermore, the elevation of reduced glutathione (GSH) levels due to increased GPx activity may contribute to cell death by accumulating a substantial amount of ROS. Antioxidant enzyme levels and their sensitive equilibrium must be maintained since they have a significant effect on cellular oxidative stress [21].

The peroxidation of polyunsaturated fatty acids in cells can lead to the generation of MDA. Increased levels of free radicals contribute to lipid peroxidation, causing an excess production of MDA. The MDA level serves as a key marker for assessing lipid peroxidation, offering valuable insights into oxidative stress [52]. Therefore, the observed rise in lipid peroxidation levels in HepG2 cells treated with Fe_3_O_4_-CS may be linked to a significant simultaneous increase in SOD activity. SOD antioxidant properties play a pivotal role in preventing lipid peroxidation. The modulation in the activities of SOD, CAT, and GPx in treated HepG2 cells could be associated with their utilization in neutralizing ROS generated during the treatment. Excessive ROS production is known to induce lipid peroxidation, involving the conversion of polyunsaturated fatty acids in the cell membrane into detrimental lipid peroxides. This process leads to membrane damage and, ultimately, cell death [56].

Table 1 shows the enzymatic activities and oxidative stress markers that were evaluated in the study. The control group exhibited SOD, CAT, GPx, and MDA levels at 54.88 U/ml, 0.09 U/L, 0.24 U/gT, and 20.08 nmol/gT, respectively. Fe_3_O_4_ nanoparticles induced notable changes in enzymatic activity with values of 173.78 U/ml for SOD, 0.07 U/L for CAT, 4.36 U/gT for GPx, and 20.42 nmol/gT for MDA. Fe_3_O_4_-CS nanoparticles displayed distinct alterations in oxidative stress markers, indicating elevated levels compared to the control and Fe_3_O_4_ groups. Notably, SOD, CAT, GPx, and MDA reached levels of 192.07 U/ml, 0.03 U/L, 18.76 U/gT, and 30.33 nmol/gT, respectively.

**Table 1:** Enzymatic Activities and Oxidative Stress Markers in HepG2 Cells Exposed to Nanoparticles

Samples	SOD (U/Ml)	CAT (U/L)	GPx (U/gT)	MDA (nmol/gT)
Control	54.88	0.09	0.24	20.08
Fe_3_O_4_	173.78	0.07	4.36	20.42
Fe_3_O_4_-CS	192.07	0.03	18.76	30.33

The observed variations in SOD, CAT, GPx, and MDA indicate an induction of oxidative stress, an increase in ROS production, and the release of antioxidant amounts following the treatment of HepG2 cells with Fe_3_O_4_-CS nanomaterials. These findings may provide valuable insights for future approaches in the design of anticancer drugs.

## Conclusion

An extensive investigation was conducted to characterize the prepared Fe_3_O_4_ nanoparticles and Fe_3_O_4_-CS using a range of analytical techniques, providing a detailed understanding of their structural, morphological, and functional attributes. FTIR spectroscopy validated the successful synthesis and coating of Fe_3_O_4_ nanoparticles with CS. The observed shifts in absorption bands indicated interactions between surface-charged Fe_3_O_4_ nanoparticles and the cationic biopolymer matrix of CS through electrostatic interactions and hydrogen bonding, resulting in the creation of a hybrid nanomaterial. Insights into the size and morphology of the nanoparticles were obtained through TEM and DLS. TEM images revealed the nearly spherical nature of both Fe_3_O_4_ nanoparticles and CS- Fe_3_O_4_, with the latter exhibiting a more uniform size distribution. DLS analysis corroborated these findings, demonstrating hydrodynamic sizes in aqueous solution consistent with TEM results. Zeta potential measurements highlighted the high stability and homogeneity of both Fe_3_O_4_ nanoparticles and CS- Fe_3_O_4_, with strongly negative values indicating repulsive forces among particles. The reduction in zeta potential following the coating with CS confirmed the successful modification of Fe_3_O_4_ nanoparticles. UV-Vis spectroscopy illustrated the formation of the nanomaterials, with absorbance shifts corresponding to the synthesis and coating processes. These spectral changes supported the surface modifications of Fe_3_O_4_ nanoparticles with CS. VSM analysis elucidated the magnetic properties of the synthesized Fe_3_O_4_ and chitosan-coated Fe_3_O_4_ nanoparticles. The saturation magnetization values indicated superparamagnetic behavior, rendering them suitable for biomedical applications such as hyperthermia cancer therapy and magnetic resonance imaging contrast agents. Biological studies delved into the cytotoxicity, DNA damage, and oxidative stress markers of the synthesized nanomaterials. The MTT assay revealed potent cytotoxicity of Fe_3_O_4_-CS against HepG2 cells, displaying a significantly lower IC50 (39.15 ± 1.7 μg/ml) compared to Fe_3_O_4_ alone (383.71 ± 23.9 μg/ml). The Comet assay demonstrated a substantial impact on DNA damage, suggesting the potential of CS- Fe_3_O_4_ to induce apoptosis and DNA double-strand breaks. Evaluation of oxidative stress markers demonstrated an increase in SOD activity after treatment with Fe3O4-CS, indicating an adaptive response to elevated ROS levels in cancer cells. Changes in ‘. and GPx activities reflected the intricate balance of antioxidant defenses in response to oxidative stress. The elevation in MDA levels indicated lipid peroxidation, emphasizing the potential of Fe_3_O_4_-CS to induce oxidative stress in HepG2 cells. Finally, the thorough characterization and biological assessments of Fe_3_O_4_ nanoparticles and Fe_3_O_4_-CS underscore their versatility and potential applications in cancer therapy. The amalgamation of magnetic properties and cytotoxic effects positions CS- Fe_3_O_4_ as a compelling candidate for further exploration in targeted drug delivery and cancer treatment.
